# Food Emulsifiers and Metabolic Syndrome: The Role of the Gut Microbiota

**DOI:** 10.3390/foods11152205

**Published:** 2022-07-25

**Authors:** Martina De Siena, Pauline Raoul, Lara Costantini, Emidio Scarpellini, Marco Cintoni, Antonio Gasbarrini, Emanuele Rinninella, Maria Cristina Mele

**Affiliations:** 1 UOC di Medicina Interna e Gastroenterologia, Dipartimento di Scienze Mediche e Chirurgiche, Fondazione Policlinico Universitario A. Gemelli IRCCS, 00168 Rome, Italy; martinadesiena@gmail.com (M.D.S.); antonio.gasbarrini@unicatt.it (A.G.); 2 UOC di Nutrizione Clinica, Dipartimento di Scienze Mediche e Chirurgiche, Fondazione Policlinico Universitario A. Gemelli IRCCS, 00168 Rome, Italy; paulineceline.raoul@policlinicogemelli.it (P.R.); marco.cintoni@gmail.com (M.C.); mariacristina.mele@unicatt.it (M.C.M.); 3 Department of Ecological and Biological Sciences (DEB), Tuscia University, 01100 Viterbo, Italy; lara.cost@unitus.it; 4 Nutrition and Internal Medicine Unit, “Madonna del Soccorso” General Hospital, 63074 San Benedetto del Tronto, Italy; emidio.scarpellini@med.kuleuven.be; 5 T.A.R.G.I.D., Gasthuisberg University Hospital, KU Leuven, Herestraat 49, 3000 Lueven, Belgium; 6 Dipartimento di Medicina e Chirurgia Traslazionale, Università Cattolica del Sacro Cuore, Largo F. Vito 1, 00168 Rome, Italy

**Keywords:** emulsifiers, gut microbiota, metabolic syndrome, obesity, polysorbates, carrageenans, insulin resistance, processed foods

## Abstract

The use of emulsifiers in processed foods and the rapid epidemic development of metabolic syndrome in Western countries over the past 20 years have generated growing interest. Evidence for the role of emulsifiers in metabolic syndrome through gut microbiota has not been clearly established, thus making it challenging for clinical nutritionists and dietitians to make evidence-based associations between the nature and the quantity of emulsifiers and metabolic disorders. This narrative review summarizes the highest quality clinical evidence currently available about the impact of food emulsifiers on gut microbiota composition and functions and the potential development of metabolic syndrome. The state-of-the-art of the different common emulsifiers is performed, highlighting where they are present in daily foods and their roles. Recent findings of in vitro, in vivo, and human studies assessing the effect of different emulsifiers on gut microbiota have been recently published. There is some progress in understanding how some food emulsifiers could contribute to developing metabolic diseases through gut microbiota alterations while others could have prebiotic effects. However, there are still many unanswered questions regarding daily consumption amounts and the synergic effects between emulsifiers’ intake and responses by the microbial signatures of each individual.

## 1. Introduction

Modern society has undergone profound socio-economic and cultural changes in recent centuries. Increased productivity and work optimization have driven the population to reduce cooking time in favor of processed food consumption. These manipulated or “ready-to-eat foods” are rich in food additives that could alter our health [1]. Emulsifiers are among the most common food additives in butter, milk, mayonnaise, sauces, ice creams, or pastries.

They play a key functional and technological role in helping processed products containing immiscible food ingredients, such as oil and water, to combine. The basic structure of an emulsifying agent includes a hydrophobic portion and a hydrophilic portion that may be either charged or uncharged. Thus, they are at the interface of water and fat, avoiding the breakdown of food ingredients, specifically by preventing separation, melting, or precipitation. The common emulsifiers include lecithins, mono- and diglycerides, polysorbates, carrageenans, guar gums, and carboxymethylcellulose. Emulsifiers are deprived of calories, proteins, lipids, and glucides. Consequently, at first sight, emulsifiers seem harmless for consumers suffering from metabolic syndrome, such as diabetics and/or obese patients. Nevertheless, recent findings showed that some emulsifiers could aggravate metabolic disorders through the modulation of gut microbiota.

Researchers have recently hypothesized that emulsifiers in processed foods may increase the translocation of bacteria through the epithelium in the gut barrier, thereby inducing inflammation. This translocation is the consequence of an increase in the permeability of the mucosa, which may be due to inflammatory lesions or disorders of the gut microbiota called dysbiosis. Increased intestinal permeability causes the translocation of lipopolysaccharide (LPS) and tryptophan-derived metabolites, subsequent metabolic endotoxemia, and chronic low-grade systemic inflammation.

Metabolic syndrome is a complex pathophysiological state with an incidence similar to a global epidemic. The World Health Organization defines metabolic syndrome as a pathological condition characterized by obesity, insulin resistance, hypertension, hyperlipidemia, and the waist-to-hip ratio [2]. Metabolic syndrome is present if three or more of the criteria mentioned above are present [3]. Metabolic syndrome also represents a risk factor for the onset of chronic diseases, including cardiovascular disease [4], type 2 diabetes mellitus [5], chronic kidney disease [6], and some types of cancer [7]. A plethora of literature data suggest the gut microbiota’s potential role in interfering with the host metabolism, thus influencing several metabolic syndrome risk factors [8,9,10].

This review presents the most common emulsifiers, detailing their functional role in processed foods and specifying the authorized acceptable daily dose intakes for consumers and maximum use levels in processed products by international food safety authorities. Secondly, an overview of recent studies focusing on food emulsifiers, gut microbiota, and the potential effect on metabolic syndrome incidence has been provided based on a systematic literature search.

## 2. Methods

A systematic literature search was performed from inception to October 2021 using three databases: Medline (via PubMed), Web of Science, and Scopus. The search query was: (microbiota” OR “microbiome”) AND (“emulsifiers” OR “carboxymethyl cellulose” OR “polysorbate 80” OR “lecithin” OR “mono- and diglycerides” OR “polysorbates” OR “carrageenan” OR “guar gum”) AND (“(metabolic AND syndrome”) OR (“insulin AND resistance”) OR “cardiovascular” OR “diabetes” OR “obesity”). We used the key term emulsifiers, and we have specified the most common emulsifiers used in processed products as mentioned by Chazelas et al. [11] and Partridge et al. [12]. Moreover, hand-searching of eligible studies was performed to check the reference lists and find original and additional references.

## 3. Emulsifiers in Processed Foods

### 3.1. Emulsifiers Classification, Safety Legislation, and Labeling: State-of-the-Art and Limitations

The Codex Alimentarius classified emulsifiers according to their functions, listing more than 260 emulsifier types [13]. On the other hand, the Food Standard Agency (FSA) lists only 63 emulsifying agents [14]. The Food and Drug Administration (FDA) of the United States lists 171 emulsifiers and emulsifying salts.

Authorities such as the European Food Safety Authority (EFSA) and the FDA require safety assessments of emulsifiers before approval for food use. In this context, an acceptable daily intake (ADI) level and maximum food additive use and exposure levels could be established [15]. A maximum use level of an additive could be defined as the highest additive concentration determined to be functionally effective in a food category. It is generally expressed as mg additive/kg of food. The maximum level of food additive emulsifier exposure combines food consumption data with food concentration data. There are three main factors involved in estimating the estimated daily intake (EDI) for a food additive: (i) frequency—how often a person daily eats a particular food containing the substance; (ii) portion size—how much of the food a person eats; and (iii) concentration—how much of the substance is in a given amount of the food. The EDI is calculated by multiplying these factors (EDI = frequency × portion size × concentration) [16].

However, to date, data on the concentrations of the emulsifiers within (ultra)processed food are lacking. Consequently, estimations of emulsifier intake can be overestimated or underestimated. An extensive database of all manufactured foods with accurate concentrations of food additives is needed to assess the population’s emulsifier exposure. Recently, a French survey estimated exposure to 13 food additives using the methods proposed in the 2001 Report from the European Commission on Dietary Food Additive Intake in the European Union [17,18]. However, comparing these results with published data, it remains challenging to draw standardized estimations due to the differences in diets between countries, methodologies, data availability, and targeted populations. Another possible approach to estimate real food additive dietary exposure is to simulate meals for adults and children, as proposed by Leclercq et al. [19], for different food additives and combine different food products in each meal. This work could be undertaken by several European countries or at an international level for other and specific diets.

The Joint Food and Agriculture Organization/World Health Organization Expert Committee on Food Additives (JECFA) represents the international scientific expert committee administered jointly by the Food and Agriculture Organization of the United Nations (FAO) and the WHO. It proposes an international numbering system to name food additives, including emulsifiers [20]. The E-number (i.e., E-171) must be indicated after having specified the technological function of the food-additive emulsifiers. The FDA imposes that all ingredients used in foods are listed by their common name. However, many manufacturers can replace the name of food additives with a number only, leading to some confusion among consumers. Moreover, none of these national authorities require the quantities of emulsifiers on food labels.

### 3.2. Common Emulsifiers Used in the Food Supply

More than 100 food-additive emulsifiers can be used in the food supply. In this part, only the main common emulsifiers have been presented [11,12].

#### 3.2.1. Carboxymethylcellulose (CMC)

Carboxymethylcellulose (CMC, E466) is a hydrocolloid having structuring, thickening, or gelling functions in the aqueous phase [21]. The food industry uses CMC in candies, chewing gums, snack foods, ketchup, and various baked goods. In the United States, CMC is listed in the FDA’s database of generally recognized as safe (GRAS) substances [22]. In Europe, CMC has been authorized as a food additive under Annex II and Annex III of Regulation (EC) No 1333/2008. The latest evaluation of enzymatically hydrolyzed CMC was conducted in 1998, and an ADI ‘not specified’ was established [23]. Following a request from the European Commission, the EFSA Panel on Food Additives and Nutrient Sources added to food delivered a scientific opinion re-evaluating the safety of CMC as a food additive [24]. The Panel concluded that there was no need for a numerical ADI and suggested an indicative daily consumption value of 660−900 mg/kg body weight per day.

#### 3.2.2. Polysorbate 80 (P80)

Polysorbate 80 (E433) acts as an emulsifier, dispersant, or solubilizer in bread, cake mix, salad dressing, shortening oil, and chocolate. According to FDA, in ice cream, frozen custard, ice milk, fruit sherbet, and non-standardized frozen desserts, the maximum amount of the additives, alone or in combination, does not exceed 0.1 percent of the finished frozen dessert [25]. Polysorbate 80 is usually added in ice creams at a 0.5% concentration to make them smoother and increase their resistance to melting [26]. The EFSA Panel recently re-evaluated the safety of polysorbates, including polysorbate as food additives. The JECFA defined an ADI of 25 mg/kg body weight/day [27].

#### 3.2.3. Lecithins

Lecithin (E322) is a natural emulsifier initially isolated from egg yolk, composed of choline, fatty acids, glycerol, glycolipids, phospholipids, phosphoric acid, and triglycerides. Today, lecithin is extracted from cottonseed, marine sources, milk, rapeseed, soybeans, and sunflower. Lecithins are mixtures or fractions of phosphatides obtained by physical procedures from animal or vegetable foodstuffs [28]. The EFSA re-evaluated lecithin as a food additive in foods for infants below 16 weeks of age and follow-up and in foods for all population groups [15]. Concerning the dietary exposure to the food additive for infants below 16 weeks of age, the maximum use levels of lecithins were defined at 260 mg/kg per day.

Sunflower lecithin can be used as an additive in food and feedstuffs [29]. Sunflower lecithin has a higher phosphatidylcholine content and lower viscosity than soy lecithin [30]. Soy lecithin has found several applications such as a fat replacer and plant-based creamer in food manufacturing [31]. Moreover, recently, the EFSA Panel authorized oat lecithin for use as a new food additive in the food category of cocoa and chocolate products [32]. The EFSA Panel concluded that there is no need for a numerical ADI.

#### 3.2.4. Propylene Glycol Alginate

Propylene glycol alginate (E405)—or propane-1,2-diol alginate—is an ester made from alginic acid (E400) and propylene glycol (E1520) derived from brown seaweed [33]. It is mainly found in salad dressings, ice cream, beer, frozen foods, bakeries, and jelly. FDA defined maximum use levels in jams and jellies as 0.4%; frozen dairy desserts, fruit and water ices, confections, and frostings as 0.5%; baked goods as 0.5%; gravies and in sweet sauces as 0.5%; gelatins and puddings as 0.6%; condiments as 0.6%; cheese as 0.9%; fats and oils as 1.1%; and seasonings and flavors as 1.7%. The EFSA defined an ADI of 55 mg/kg body weight per day [34].

#### 3.2.5. Carrageenans

Carrageenan’s (E407) use as a food additive in the Western diet has increased substantially over the last 50 years [35]. Indeed, carrageenan acts to thicken, stabilize, and emulsify a wide variety of foods, including chocolate milk, ice cream, cottage cheese, sour cream, processed meats, and mayonnaise [36]. Carrageenan also improves the texture of infant formulas, dairy products such as yogurt, milk alternatives such as almond milk, processed meats, and soy-based products [37]. Carrageenan can act like fat, making it particularly attractive for consumers increasingly preferring low-calorie foods. Carrageenan also serves as an alternative to gelatin for vegan and vegetarian products. Estimates regarding the ADI of carrageenan vary from 20 to 200 mg/day [38]. The EFSA has established a temporary ADI of 75 mg per kilogram of body weight [39]. At the same time, in the United States, carrageenans are permitted by the FDA for use as a food additive when used in the amount necessary for the intended effect.

#### 3.2.6. Gums (Acacia, Arabic, Xanthan, Guar)

Acacia gum (E414) is a natural emulsifier, stabilizer, texturizer, and source of fiber [40,41]. Acacia gum is unlikely to be absorbed intact and is slightly fermented by intestinal microbiota [42]. The oral daily intake of a large amount of acacia gum, up to 30,000 mg acacia gum/per person per day for up to 18 days, was well-tolerated in adults. There is no need for a numerical ADI and no safety concern for acacia gum. Arabic gum is used in the food industry as a stabilizer, emulsifier, and thickening agent in icing, fillings, soft candy, chewing gum, and other confectionery and to bind the sweeteners and flavorings in soft drinks [42,43].

Xanthan gum is a high molecular weight polysaccharide produced by a pure culture fermentation of a carbohydrate with strains of *Xanthomonas campestris*. At the concentration of 1%, xanthan gum can significantly increase the viscosity of a liquid [44]. In foods, xanthan gum is found in salad dressings and sauces. There is no need for a numerical ADI, and there is no safety concern for xanthan gum (E415) [45].

Guar gum (E412) is a gel-forming galactomannan extracted from a leguminous plant [46]. Guar is a polysaccharide with one of the highest molecular weights of naturally occurring water-soluble polymers [46]. Guar gum is commonly found in ice cream, yogurt, salad dressing, gluten-free baked goods, sauces, kefir, and breakfast cereals [47]. Guar gum enhances the consistency of tomato ketchup more prominently than CMC, sodium alginate, gum acacia, and pectin. In addition to guar gum, serum loss and flow values of tomato ketchup decrease, making it a novel thickener for tomato ketchup [48,49]. In 2018, the EFSA panel re-evaluated the safety of guar gum [50]. Oral intake of guar gum was well tolerated in adults. There is no need for a numerical ADI for guar gum. However, for its use in foods intended for infants and young children, abdominal discomfort should be monitored.

#### 3.2.7. Maltodextrin

Maltodextrins are saccharide polymers consisting of D-glucose units linked primarily linearly with alpha-1,4 bonds, but they can also have a branched structure through alpha-1,6 bonds [51]. Their functional properties are closely related to their low-sweetness composition. The reducing sugar composition in maltodextrin will affect its sweetness, viscosity, and other properties. According to FDA, maltodextrin can be a GRAS used in food with no limitation other than current good manufacturing practices [52].

#### 3.2.8. Agar Agar

Agar agar (E406) is a hydrocolloid commonly used as a gelling agent and thickener in food. It is the first used phycocolloid, much earlier than alginates and carrageenan, which are also extracted from (marine algae) seaweed. It is suitable for vegetarian products since it substitutes gelatin derived from animal skin and bones. Agar agar is mainly found in jelly, bakery, confectionery, dairy products, beverage, and meat products. FDA has also defined maximum use levels in various food products: (i) 0.8% in baked goods and baking mixes; (ii) 1.2% in soft candy; and (iii) 0.25% in all other food categories [53]. The EFSA concluded that there is no need for a numerical ADI and that there is no safety concern for the agar.

#### 3.2.9. Glycerol Monolaurate

Glycerol monolaurate (GML, E471) is used as a natural, effective antibacterial emulsifier in the food industry [54]. GML is a monoglyceride of C12:0, occurring naturally in breast milk and coconut oil. GML has been used as an antimicrobial and antiviral emulsifier and is defined as GRAS by the US Food and Drug Administration. It is found in numerous processed foods such as processed cakes, bread, and ice creams.

#### 3.2.10. Rhamnolipids and Sophorolipids

Rhamnolipids and sophorolipids are two biotechnological emulsifiers of microbial origin. Their emulsifying properties are associated with these molecules’ amphiphilic character, allowing them to accumulate between fluid phases and reduce surface and interfacial tensions. Due to their non-toxicity, they represent novel, potentially food-safe emulsifiers. Indeed, they have a natural origin source (biotechnological production from renewable resources), thereby qualifying them as adequate alternatives for emulsifiers of chemical origin. Rhamnolipids and sophorolipids [55,56] are also classified as biodegradable biosurfactants. They can be found in numerous ultra-processed foods such as bread, hamburger, baguettes, pizza, croissants, salad dressing, bread, cakes, biscuits, and ice cream to improve their stability and shape, structure, and texture [57].

To sum up, this part highlights the different technological roles of the emulsifiers in processed foods and their omnipresence in many everyday food products. Only for some emulsifiers have the ADI and maximum acceptable daily exposure been indicated by food safety authorities. Thus, most emulsifiers do not have daily quantity restrictions. Although many emulsifiers are consumed daily, to date, their cumulative quantities and the synergic effects of each other have not yet been assessed. Moreover, only the presence of emulsifiers, and not the amount, is reported on food labels; therefore, it has been impossible to estimate actual emulsifier intakes. These statements are in opposition to the growing scientific interest in this type of food additive in terms of gut microbiota modulation and their possible associations with the growing incidence of metabolic syndrome. In the next paragraphs, we focus on the associations between metabolic syndrome and gut microbiota variations (dysbiosis) and detail the recent findings assessing the relationships between gut microbiota, metabolic syndrome, and some dietary emulsifiers

## 4. Gut Microbiota and Metabolic Syndrome

The rapid social and environmental transformation affecting our planet has subverted food consumption by affecting lifestyle. The daily timetable has changed in modern society, and less attention is paid to food. The habit of “eating out” has spread and impacted people’s choices and tastes. There has been increased consumption of processed, industrial, and ready-to-eat foods with lower nutritional properties than healthy food. Some fundamental components such as olive oil, fruits, and vegetables are consumed less and replaced by diets rich in fats and proteins with quality aspects that are often lacking. This has led to a dramatic increase in metabolic-disruption-related diseases, causing obesity, type 2 diabetes, cardiovascular disease, and metabolic syndrome.

Metabolic syndrome is a set of altered physical conditions such as fatty accumulation, insulin resistance, elevated blood pressure, and hepatic steatosis that significantly increase the risk of severe cardiovascular and/or ischemic disease. Although the precise cause remains unknown to date, genetic, environmental, and dietary factors are implicated in the onset of this syndrome. The microbiome has recently conquered a key role, and numerous studies have confirmed its relevance in metabolic disease progression. The analysis conducted by Ferrer et al. [58] on fecal samples from obese adolescents showed a strong imbalance between Bacteroidetes and Firmicutes after comparison with healthy controls. In obese subjects, the Firmicutes phylum was found to be much more abundant (94.6%) while, in contrast, the Bacteroidetes phylum showed a low concentration (3.2%).

In contrast, this remarkable difference was not found in normal-weight adolescents. The gut microbiome can influence metabolic syndrome through the production of SCFAs. SCFAs, mainly acetate, propionate, and butyrate, promote the regeneration and protection of intestinal cells, mucin production, reduction in hypercholesterolemia levels, and the release of hormones and/or neurotransmitters important for the regulation of intestinal motility and insulin resistance. Through the production of the hydrolysis enzyme for bile acids, particular bacterial species facilitate their conversion to the salified form, which, in turn, allows the breakdown of fats, thereby reducing serum cholesterol levels.

Anti-obesity effects from the combined administration of probiotics and prebiotics have been obtained from both in vivo and clinical studies. Microbiota modulation through diet, prebiotics, and probiotic supplementation may play a role in the treatment of the metabolic syndrome. Combined *Bifidobacteria* and/or *Lactobacilli* administration would appear to have synergistic effects in vivo models [59].

Endotoxemia related to dysbiosis and increased intestinal permeability has been found to be an additional risk factor for insulin resistance, as revealed in murine studies, and therefore, probiotics, by suppressing endotoxemia, have a protective effect. LPS are some of the pro-inflammatory metabolites produced by certain bacterial species, which, upon entering circulation due to altered intestinal permeability, go on to stimulate endocannabinoid receptors, aggravating the state of obesity.

Exercise can attenuate dysbiosis and intestinal permeability and increase the abundance of butyrate-producing bacteria. In animal models of obesity, butyrate has been seen to increase energy expenditure, reduce adiposity, improve insulin sensitivity, and stimulate the production of satiety hormones.

Another suspected risk factor for metabolic syndrome is chronic stress, which can alter the microbiome in important ways. It turns out that the microbiome influences cortisol levels. Recent clinical studies showed that manipulating the microbiome’s composition in favor of *Bifidobacteria* could reduce the physiological risk of metabolic syndrome in overweight adults [60,61].

## 5. Relationship between Emulsifiers and Metabolic Syndrome through Gut Microbiota

The maintenance of body homeostasis is an adaptive process that has developed over millennia and still represents a brilliant form of evolution. From birth throughout life, billions of molecules are ingested daily with food and interact with our gut. In healthy individuals, complex networks are established between our systems and the external environment to safeguard homeostasis. Mucus, intestinal cells (IECs), tight junctions, and resident microbiota compose the functional intestinal barrier that cooperates with the immune system to discriminate between harmful and not harmful matter [62]. In this dynamic process, the gut microbiota plays a key role, intended as the wide range of microorganisms colonizing our gut, including bacteria, viruses, protozoa, and fungi. Common diseases such as metabolic syndrome, diabetes, hypertension, obesity, and cardiovascular disease are associated with qualitative and quantitative gut microbiota alterations. Several studies suggest that dietary emulsifiers, such as CMC, polysorbate 80 (P80), carrageenans, and gums could shift the gut microbiota composition to a proinflammatory pattern predisposing to metabolic syndrome and intestinal inflammatory disease [63,64]. Emulsifiers are frequently non-absorbed compounds that directly interact with the gut microbiota in our intestinal lumen. We discuss the main studies showing a potential relationship between emulsifiers and metabolic syndrome through the gut microbiota.

### 5.1. In Vitro Studies

Dietary emulsifier exposure can shift the gut microbiota, inducing a proinflammatory signaling, predisposing the body to several diseases. Using a human microbiota in vitro model—the MiniBioReactor Arrays (MBRA)—Naimi S et al. [65] evaluated the impact of 20 dietary emulsifiers on the microbiota. The study showed how the administration of commercially available emulsifiers alters microbiota composition and gene expression, predisposing the body to a chronic inflammatory state. In particular, the administration of polysorbate 80, carrageenan, and agar agar could reduce the relative abundance of Clostridiales order (especially *Faecalibacterium* genus) and *Verrucomicrobiales*, driven by the *Akkermansia* genus well-known for its anti-inflammatory properties. Moreover, xantham gum, sorbitan monostearate, glyceryl stearate, and carrageenan administration in vivo increase bioactive lipopolysaccharide (LPS) levels and encourage flagellin expression [66]. Maltodextrin, which has emulsifying properties, has a direct effect on gut microbiota, enhancing cell adhesion and biofilm formation through the stimulation of multiple E. coli strains [67].

An interesting in vitro study conducted by Miclotte et al. [68] investigated the effect of five dietary emulsifiers on fecal microbiota from 10 human individuals upon 48 h of exposure. In particular, two mainstream chemical emulsifiers (CMC and P80), a natural extract (soy lecithin), and biotechnological emulsifiers (sophorolipids and rhamnolipids) have been compared. Microbial profiling analysis was performed, and short-chain fatty acid (SCFA) levels were measured to evaluate how exposure to different dietary emulsifiers affects the general microbial metabolic activity. The food industry would like to offer them as healthier than chemical emulsifiers. However, their strong antimicrobial properties and their effects on gut microbiota should be considered and well-analyzed before their application in food industries. Compared to soy lecithin, CMC, P80, rhamnolipids, and sophorolipids significantly drop microbiota diversity indices. Indeed, data analyses showed an increase in pathogenic *Escherichia*/*Shigella* and *Fusobacterium* with respective decreases in beneficial Bacteroidetes after in vitro rhamnolipids and sophorolipids exposure.

Furthermore, studies showed an emulsifier-dependent shift in SCFA profiles, such as increased propionate production and a reduction in butyrate up to 96% compared to controls. Changes in the mucus barrier upon exposure to CMC and P80 were analyzed. In mucus-producing cell cultures, slower E. coli speed and particle diffusion rates through mucus were observed after emulsifiers’ administration. CMC and P80 impact the intestinal barrier and structural properties of the mucus, which may contribute to the development of intestinal inflammation.

The impact of CMC and P80 on gut microbiota was also analyzed with the mucosal simulator of the human intestinal microbial ecosystem (M-SHIME) [64]. This model maintains a complex stable human microbiota in the absence of a live host. Administration of these emulsifiers directly affects the human microbiota. When transplanted to germ-free mice, CMC-treated and P80-treated M-SHIME suspensions promote low-grade inflammation, indicating that the direct effects of these emulsifiers on the microbiota are sufficient to drive low-grade inflammation and metabolic disease. Moreover, CMC altered microbiota gene expression with increased bioactive flagellin levels only after one day.

### 5.2. Animal Studies

CMC and polysorbate significantly modify microbiota diversity, increasing Proteobacteria and *Escherichia coli* levels and reducing *Bacteroides* and *Clostridia* [64]. Furthermore, these compounds administrated in animal models increased the circulating LPS levels and flagellin expression, promoting inflammation and determining morphological and functional changes in the intestinal barrier. CMC stimulates the intestinal bacteria to become more virulent, enhancing their motility and ability to colonize the epithelium. Microorganisms became more adherent to the intestinal mucosa and migrated deeply into the crypts [69]. Indeed, alterations in crypt depth and intestinal villi length have been observed. Animal models showed how P80 indirectly reduced mucus thickness via microbiota alterations. The pivotal role of the intestinal microbiota in this dysfunctional process has been demonstrated in germ-free mice. The administration of P-80 could not modify the viscosity of the intestinal barrier mucus [70]. Despite being classified as safe emulsifiers, carrageenan could disrupt the intercellular junctions acting on actin filament and the zonula occludens-1 [Z0-1] proteins between intestinal cells [71]. These modifications predispose the body to leaky gut conditions and activate the proinflammatory pathway, characteristic of metabolic syndrome [72].

Phthalic acid esters (PAEs) are used as plasticizers in food packaging. Phthalate (DEHP) is the most widely used and could contaminate our diets. Polysorbate 80 promotes the intestinal absorption and bioavailability of di-2-ethylhexyl phthalate (DEHP) and its primary toxic metabolite mono-2-ethylhexyl phthalate (MEHP) by disrupting the intestinal barrier through gut microbiota modifications. Alterations in the intestinal barrier after P80 administration in rats were studied with 5-aminofluorescein-MEHP (MEHP-AF), a small fluorescent molecule used as a tracker of the toxic metabolite mono-2-ethylhexyl phthalate [73]. If we consider that processed foods are often stored in packaging containing the molecules mentioned above, we realize the impact that processed foods could have on our health.

In genetically predisposed mice, emulsifiers stimulate bacterial overgrowth, aggressive ileitis, and colon inflammation, similar to inflammatory bowel diseases (IBD). Zangara et al. [74] confirmed that food additives such as maltodextrin and CMC may lead to IBD in predisposed mice. They showed alterations in the gut microbiome and intestinal barrier after emulsifiers’ administration in an interleukin-10-deficient (IL10KO) mice model (genetically predisposed to colitis).

Dysbiosis and intestinal barrier impairment may predispose the body to chronic inflammation, obesity, metabolic syndrome, and Nonalcoholic Fatty Liver Disease (NAFLD), as reported in murine models after the administration of dietary emulsifiers. Singh et al. [75] showed a close relationship between the administration of P80 and alterations in the gut microbiota’s composition, contributing to the development of chronic inflammatory disease and NAFLD. Compared to control mice, P80-fed mice showed markers of metabolic syndrome such as altered glycemic tolerance, hyperinsulinemia, and increased levels of liver enzymes such as alkaline phosphatase (ALP), aspartate aminotransferase (AST), and alanine aminotransferase (ALT), suggesting biliary and hepatocellular damage. Significant weight gains and a marked increase in adipose tissue (measured by fat mass) were also observed. Increased levels of *Helicobacter*, *Campylobacter jejuni*, and *Salmonella* correlated with these damages, suggesting a detrimental role of microbiota in the pathogenesis of the disease.

GML has dose-dependent effects on the gut microbiota, glucose, lipid metabolism, and inflammatory response in mice models. Regardless of the dosages, GML administration induced body weight gain in mice. Low-dose dietary GML in low-fat diet (LFD)-fed mice could also cause low-grade systematic inflammation and gut microbiota dysbiosis [76]. However, high-dose GML supplementation in mice, targeting the gut microbiota, could attenuate the high-fat diet (HFD)-induced metabolic disorders [77]. The administration of 450 mg kg−1 of GML in HFD-fed mice impacts lipid metabolism, improving metabolic syndrome. Other effects have been observed in mice fed a high dose of GML (1600 mg kg−1), such as reduced levels of serum proinflammatory cytokine (TGF-β1 and IL-22) [78]. In addition, GML improves the intestinal epithelium structure, decreasing hyperlipidemia, visceral fat deposition, and the size of epididymal adipocytes. These effects ameliorate insulin sensitivity and systematic inflammation in animal models. Mice fed with GML show a decreased abundance of anti-inflammatory *Akkermansia*, *Bifidobacterium*, and *Lactobacillus* and increased levels of *Escherichia coli*, *Lactococcus*, and *Flexispira*. GML presents antibacterial and antiparasitic properties. It reduces the abundance of pathogenic bacteria and interferes with parasite multiplication in the intestine. In addition, GML increased immunoglobulin and protein serum levels, strengthening the broiler’s immune system [78]. For these reasons, GML is considered a growth promoter, and it is used as a feed supplement in poultry production to stimulate broiler feed performance and weight gain [79]. In broilers, GML acts on gut microbiota, raising the levels of acid-producing bacterial groups such as *Lachnospiraceae*, *Christensenellaceae*, and *Blautia*, with consequent higher production of SCFAs (butyrate, propionate, valerate, and isovalerate) [80].

### 5.3. Human Studies

Emulsifiers inducing gut microbiota dysbiosis can stimulate chronic inflammation and predispose the body to the development of metabolic syndrome in in vitro studies and animal models. However, the impact of their administration on human metabolism remains to be understood. Indeed, emulsifier consumption leads to metabolic endotoxemia, which is associated with chronic diseases such as metabolic syndrome. However, the grade of metabolic endotoxemia is dependent on the commensal or pathogenic microorganism that produces LPS and, consequently, on LPS structure. This explains why the LPS structure and, therefore, the immune response, is different for animals and humans due to their differences in microbial species that vary across diet, lifestyle, and animal species [81], often overlooked in preclinical and in vivo studies.

Chassaing et al. performed a randomized, double-blind, controlled-feeding study. Healthy adults consumed only emulsifier-free diets or an identical diet enriched with 15 g of CMC per day for 14 days [82]. This dosage of CMC is higher than the daily consumption of most of the population but reflects the consumption of persons whose diets are comprised mainly of highly processed foods. Blood, plasma, urine, and fecal samples were collected, and a panel of inflammatory and metabolic parameters was analyzed to characterize the metabolic syndrome. In addition, oral glucose tolerance testing was performed to test insulin sensitivity. Data analyses found that adding CMC to a healthy additive-free diet increased postprandial abdominal discomfort and altered intestinal microbiota composition. In addition, CMC reduced microbiota richness and diversity, and decreased *Faecalibacterium prausnitzii* and *Ruminococcus* spp. and increased levels of *Roseburia* sp. and *Lachnospiraceae* were observed. In contrast to what has been observed in in vitro and animal models, fecal levels of lipopolysaccharide and flagellin were not affected by CMC consumption.

As previously discussed, an integer intestinal barrier is fundamental for maintaining homeostasis. Alterations in mucus layer thickness or strength of the tight junction could induce chronic inflammation leading to leaky gut and increased intestinal permeability. The translocation of microorganisms from the intestinal lumen into the circulatory stream could activate immunological networks. Disruption of the intestinal barrier induces a dysfunctional state characterized by chronic inflammation, which underlies many diseases, including metabolic syndrome. A randomized controlled crossover study analyzed how the impact of optimizing fat structure in meal can be a dietary strategy to lower the metabolic effect of postprandial endotoxemia in obese men [83]. Endotoxemia is a gut-derived condition that occurs after the consumption of energy-rich meals. It is characterized by a transient increase in circulating pro-inflammatory LPS, and it is one of the driving mechanisms of inflammation in metabolic syndrome. In their pilot study, Vors et al. [83] assumed that lipid absorption can be modulated by emulsifying dietary fat and suggested an impact of fat structure in the meal on the postprandial absorption in obese men. Emulsifying dietary fat induces a specific dynamic response of postprandial LPS bound to chylomicrons that seem more prone to clearance than spreading fat.

Several studies found a positive association between the consumption of ultra-processed food and the likelihood of developing metabolic syndrome. A cross-sectional survey of 789 40- to 70-year-old subjects confirmed an independent dose-response association between ultra-processed food and metabolic syndrome [84]. The significantly higher odds for presumed NASH among subjects with NAFLD who consumed high doses of ultra-processed food were even more striking. NASH is characterized by hepatocellular injury, chronic inflammation, and a higher risk of end-stage liver diseases such as cirrhosis and liver cancer. A positive association between an ultra-processed food diet and a single components of metabolic syndrome such as hypertension, dyslipidemia (low HDL levels and hypertriglyceridemia), and impaired fasting glucose were found [85].

**Figure 1:**
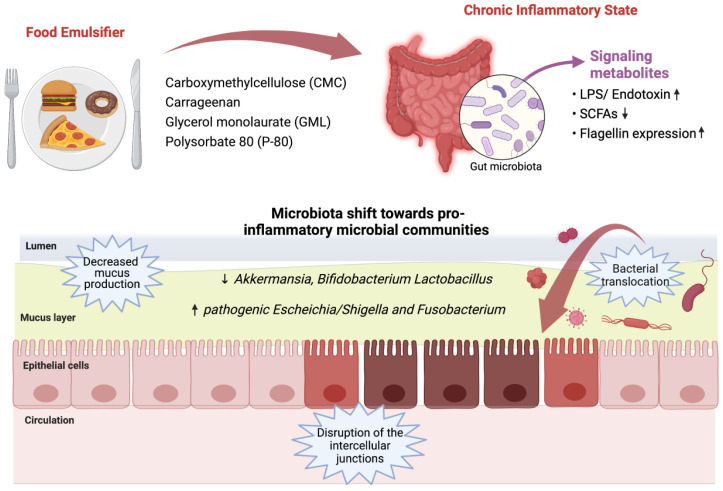
The impact of some common food emulsifiers on gut microbiota and intestinal barrier. Abbreviations: LPS, lipopolysaccharide; SCFAs, short-chain fatty acids; ↓ decrease; ↑ increase.

Figure 1 highlights the effect of some common food emulsifiers on gut microbiota and intestinal barrier. All these observations suggest that the use of processed foods rich in emulsifiers, characteristic of a modern (Western) diet, may increase the incidence of metabolic syndrome and other chronic inflammatory diseases. 

## 6. Not All So Bad: Emulsifiers to Date Considered Safe

Not all emulsifiers should be considered equal. Recent studies showed that some compounds might even have beneficial effects on our organism, such as reducing lipid oxidation. Some surfactants, phospholipids, proteins, polysaccharides, and colloidal particles explicate these antioxidant properties by stabilizing lipid droplets and preventing them from aggregating. There is contrasting evidence about the effects of GML in humans. Some studies characterized this compound as a “food-safe” emulsifier, demonstrating how GML high-dose supplementation in mice, targeting the gut microbiota, could attenuate high-fat diet (HFD)-induced metabolic disorders. Administration of 450 mg kg^−1^ of GML in HFD-fed mice impacts lipid metabolism, improving metabolic syndrome. Other effects observed in high-dose GML-fed mice have reduced levels of serum proinflammatory cytokines and circulating LPS, improvement in intestinal epithelium structure, and decreasing hyperlipidemia, visceral fat deposition, and the size of epididymal adipocytes. These effects ameliorate insulin sensitivity and systemic inflammation in animal models [86].

GML is used to stimulate broiler feed performance and weight gain [87]. GML presents antibacterial and antiparasitic properties. It reduces the abundance of pathogenic bacteria, interferes with parasite multiplication, and increases immunoglobulin and protein serum levels, strengthening the broiler’s immune system [87]. In broilers, GML acts on gut microbiota, raising the levels of the acid-producing bacterial groups *Lachnospiraceae*, *Christensenellaceae*, and *Blautia*, with consequent higher production of SCFAs (butyrate, propionate, valerate, and isovalerate). The question spontaneously arises: does GML alter the meat we eat? If we consider the organoleptic point of view, the answer is no, but if we consider the meat’s chemical composition and nutritional properties, the answer could be yes. Studies showed significantly lower lipids levels in the meat of broilers fed with glycerol monolaurate compared with controls. Supplementation with GML in broiler diets can influence nutritional characteristics of meat in a concentration-dependent manner. Reduced levels of health-damaging saturated fatty acids (SFA) and increased levels of health-beneficial polyunsaturated fatty acids (PUFA) were observed. Instead, there were no differences between groups in terms of percentage of total monounsaturated fatty acids [88]. Moreover, GML has a direct and indirect antioxidant action decreasing lipid peroxidation in meat. GML supplementation in broiler increased PUFA concentrations of the meat that we consumed. Foods containing PUFAs, particularly n-3 fatty acids (eicosapentaenoic acid, docosahexaenoic acid, and α-linolenic acid), protect us against the development of various cardiovascular and neurological diseases. GML’s effects on gut microbiota are still unclear, and further studies are needed to better understand GML’s effects on human health.

Agar agar shows anti-inflammatory, antioxidant, and immunomodulatory properties in vivo in the model of male *Drosophila*. Nutritional addition of this marine prebiotic improved fruit flies’ survival rate, decreasing the damage of epithelial cells in midgut [88], improving the immune capacity by upregulating the AMP expression, and suppressing the excessive autophagy by activating the TOR and AMPK pathways. Furthermore, supplementation with agar oligosaccharides decreased the abundance of *Klebsiella* [89], a well-known pro-inflammatory bacterium with consequent reduction of the inflammatory stress. A recent study investigated two acacia gums found in commercial dietary fiber drinks [90]. Acacia gum significantly promotes *Bifidobacteria* production and inhibits the proliferation of the *Clostridium histolyticum* group, commonly identified as a marker of gut dysbiosis. Acacia gum could positively promote the modulation of microbial species and SCFA production, especially butyrate [91].

Interestingly, a recent mice model study investigated the effect of rapeseed lecithin and soy lecithin on gut microbiota [92]. The consumption of lecithin significantly increased the fecal abundance of *Clostridium leptum*, regardless of origin or dose. The *Clostridium leptum* group, including *Faecalibacterium prausnitzii* and certain species of *Eubacterium* and *Ruminococcus*, and the *Clostridium coccoides* group, including *Roseburia intestinalis*, are butyrate-producing bacteria [93]. Moreover, rapeseed lecithin increased postprandial essential α-linolenic acid abundance and induced beneficial modifications in the bile acid profile. Rapeseed lecithin may then appear as a promising natural emulsifier.

Table 1 illustrated the main findings of in vitro, in vivo, and human studies about the associations of food emulsifiers with composition and functions of the gut microbiota.

## 7. Conclusions and Perspectives

This review gathers current knowledge on the safety, legislation, and manufacturing use of emulsifiers commonly found in processed foods, opening the debate on the lack of assessment of the impact of cumulative intake of food additives and the potential ‘cocktail’ effects/interactions of mixtures. Furthermore, currently, only the presence of emulsifiers, and not their quantity, is reported on food labels, making it impossible to estimate emulsifier intakes. Moreover, food emulsifiers without a specified ADI can be used without limitations other than current good manufacturing practice. Probably, daily emulsifier intakes are currently underestimated, especially in Western countries.

In parallel, the number of in vitro, animal, and human studies is growing to show how certain emulsifiers could contribute to developing metabolic and inflammatory diseases through the modulation of the gut microbiota. Once ingested through processed food, emulsifiers dynamically interact with the intestinal microbiota within the lumen of the gastrointestinal tract. The microbiota shift towards pro-inflammatory microbial communities, and the alterations in the intestinal barrier after administration of emulsifiers trigger a chronic inflammatory state that determines bacterial translocation within the circulatory stream and predisposes the body to the development of metabolic diseases. On the contrary, other emulsifiers could have prebiotics effects of improving and preventing gut dysbiosis and metabolic disorders. It also remains to be clarified whether the gut microbial characteristics of each individual play a role in response to food emulsifier exposure and what is the impact of the same emulsifier in the same amount in individuals with different microbiota signatures.

## Figures and Tables

**Table 1:** Dietary Emulsifiers and their associations with composition and functions of the gut microbiota in in vitro, in vivo, and human studies.

Emulsifier Name	E-Number	Origin	Foods	ADI (per kg of Body Weight per Day)	Effects on Gut Microbiota and Metabolic Health		
					In Vitro Studies	Animal Studies	Human Studies
Agar Agar	E406	Natural	Jelly, bakery, confectionery, dairy products, beverage, meat products	No ADI	↓ *Clostridiales* (*Faecalibacterium* genus) ↓ *Verrucomicrobiales* [65]	↓ *Klebsiella* [90] ↑ *Gluconobacter* [89] -antiaging -anti-inflammatory [89]	
Carboxymethylcellulose	E466	Artificial	Desserts, snacks, edible ices, chewing gums, vegetable oil, breakfast cereals, food supplements, creams, milk products, dried fruit, nut butter, chocolate products, bread and rolls, processed cheese, sauces, soups, meat products	No ADI Maximum use levels (per kg body weight per day): 660−900 mg	↓ Microbiota diversity indices [68] Changes in mucus barrier [68] Altered microbiota gene expression with increased bioactive flagellin levels [64]	Modify microbiota diversity ↑ *Proteobacteria* ↑ *Escherichia coli* ↓ Bacteroides ↓ Clostridia ↑ LPS levels ↑ flagellin expression ↑ Increased pro-inflammatory potential	↓ Microbiota richness ↓ Microbiota diversity ↓ *Faecalibacterium prausnitzii* [82] ↓ *Ruminococcus spp*. ↑ *Roseburia spp.* and *Lachnospiraceae* [82] No variations in fecal levels of LPS and flagellin [82]
Carrageenan	E407	Natural	Dairy products, chocolate milk, ice cream, cottage cheese, sour cream, processed meats, mayonnaise, infant formulas, almond milk, processed meats, soy-based products, vegan and vegetarian products	75 mg	↑ LPS levels [66] ↑ flagellin expression [65]	↓ *Clostridiales* (*Faecalibacterium* genus) ↓ *Verrucomicrobiales* [66]	Disrupt the intercellular junctions acting on actin filament and the zonula occludens-1 [Z0-1] proteins between intestinal cells [71]
Glycerol monolaurate	E471	Natural	Processed cakes, bread, and ice creams	No ADI	Induced body weight gain [76] Impact lipid metabolism improving metabolic syndrome in HFD [94] ↓ LPS in high-fat mice [78] Worsen lipid metabolism in LFD [78] ↓ *Akkermansia*, *Bifidobacterium*, and *Lactobacillus* [76] ↑ *Escherichia coli*, *Lactococcus*, and *Flexispira* [76]		
Gums	E414 acacia gum E412 guar gum E415 xanthan gum	Natural	Ice creams, yogurt, salad dressing, gluten-free baked goods, sauces, and breakfast cereals	No ADI	Acacia gum: -significantly promotes *Bifidobacteria* proliferation—inhibits the *Clostridium histolyticum* group [90] -decrease gut dysbiosis, -↑ SCFA production, especially butyrate [90]		
Lecithins	E322	Natural	Cocoa and chocolate products, margarine, biscuits and pastries, confectionery, baby food	No ADI		↑ *Clostridium leptum* (butyrate production bacteria) Anti-inflammatory effects [92]	
Maltodextrin	-	Artificial	Cooked cereals, rice, meat substitutes, bakery foods, salad dressings, frozen meals, soups, sweets, energy, and sports drinks	No ADI			↑ *Bifidobacterium* [91]
Polysorbate 80	E433	Artificial	Ice creams, whipped toppings, and other frozen desserts	25 mg	↓ *Clostridiales* (*Faecalibacterium* genus) ↓ Verrucomicrobiales ↓ Microbiota diversity indices [68] Changes in mucus barrier [68]	↓ microbiota diversity, increasing *Proteobacteria* and *Escherichia coli* levels and reducing *Bacteroides* and Clostridia [64] ↑ LPS levels ↑ flagellin expression [64] Microbiota encroachment, altered species composition, increased pro-inflammatory potential [69]	Altered glycemic tolerance, hyperinsulinemia, increased levels of liver enzymes as alkaline phosphatase (ALP), aspartate aminotransferase (AST), and alanine aminotransferase (ALT) [75] ↑ body weight [75] ↑ adipose tissue [75]
Propylene glycol alginate	E405	Artificial	Dried soups, salad dressings, cakes, muffins, biscuits, cupcakes, powdered drink mixes, soft and alcoholic drinks	55 mg			
Rhamnolipids and Sophorolipids	-	Natural	Bread, hamburgers, baguettes, pizza, croissants, salad dressing, bread, cakes, biscuits, and ice creams	No ADI	↑ pathogenic *Escherichia/Shigella* and *Fusobacterium* ↓ Bacteroidetes [68] ↑ flagellar assembly and general motility [68] ↓ SFCAs production (especially butyrate and propionate) [68]	-	-

Abbreviations: ADI, Acceptable Daily Intake; HFD, High-Fat Diet; LFD, Low-Fat Diet; LPS, Lipopolysaccharide; SCFA, Short-Chain Fatty Acid; ↓ decrease; ↑ increase.

## Data Availability

Not applicable.
